# Comparison of current treatment strategy for osteonecrosis of the femoral head from the perspective of cell therapy

**DOI:** 10.3389/fcell.2023.995816

**Published:** 2023-03-22

**Authors:** Jiaqian Wang, Peirong Xu, Liang Zhou

**Affiliations:** ^1^ Department of Orthopaedic, Wuxi No 5 People's Hospital, Wuxi, Jiangsu, China; ^2^ Department of Orthopedics, Lianshui County People’s Hospital, Huai’an, Jiangsu, China

**Keywords:** osteonecrosis of the femoral head, core decompression, bone grafting, bone marrow transplantation, bone marrow dose, cell number

## Abstract

**Aims:** The purpose of our study is to compare the effects of core decompression (CD) and bone grafting (BG) on osteonecrosis of the femoral head (ONFH). And evaluate the efficacy of CD based on cell therapy to provide guidance for the dose and number of cells.

**Methods:** We searched PubMed, Embase, and the Cochrane Library between 2012 and 2022, with keywords including “osteonecrosis of the femoral head”, “core decompression” and “bone grafting”. We selected comparative studies of CD and BG, and the comparison of CD combined with bone marrow (BM) transplantation and CD alone. Changes in hip pain were assessed by VAS, hip function were assessed by HHS and WOMAC, and THA conversion rate was used as an evaluation tool for femoral head collapse. From these three aspects, the dose of bone marrow and the number of cells transplantation were subgroup analyzed.

**Results:** Eleven studies were used to compare the efficacy of CD and BG. There was no significant difference in HHS, and the THA conversion rate of BG was significantly lower than that of CD. Thirteen CD studies based on cell therapy were included in the meta-analysis. Bone marrow aspiration concentrate (BMAC) can significantly improve VAS (mean difference (MD), 10.15; 95% confidence intervals (CI) 7.35 to 12.96, *p* < 0.00001) and reduce THA conversion rate (odds ratio (OR), 2.38; 95% CI 1.26 to 4.47, *p* = 0.007). Medium dose bone marrow fluid has a lower *p*-value in THA conversion rate. The *p* values of bone marrow mononuclear cells (BMMC) of 10^9^ magnitude in VAS score were lower.

**Conclusion:** In general, there is no consensus on the use of BG in the treatment of ONFH. The enhancement of cell-based CD procedure shows promising results. Using 20 mL BMAC and 10^9^ magnitude BMMC is likely to achieve better results.

## Introduction

Osteonecrosis of the femoral head (ONFH) is a common orthopedic disease that causes bone tissue necrosis due to damage or interruption of blood supply, and then leads to structural changes of femoral head, resulting in hip pain and dysfunction (Mont et al., 2006). Total hip arthroplasty (THA) is still the first choice for the treatment of advanced-stage femoral head collapse, especially secondary hip arthritis (Capone et al., 2017). However, for young patients, the best goal of treatment is to preserve (rather than replace) the intact femoral head. The effective treatment of early ONFH is still a difficult problem in the field of orthopedics, and various treatment methods have not reached a consensus (Roth et al., 2018). Currently, it is considered that non-operative treatment is usually ineffective in preventing progression (Mont et al., 2010). In the early stage, various joint preservation operations should be tried to prevent the collapse of the femoral head (Mont et al., 2020).

For pre-collapsed femoral head, core decompression (CD), bone grafting (BG), osteotomy and tantalum rod are several mainstream surgical methods. Due to the increased incidence of complications in patients undergoing THA after tantalum rod failure and the difficulty of conversion of THA after osteotomy, these two methods are not commonly used (Olsen et al., 2016; Osawa et al., 2017). However, for pre-collapsed femoral head, the choice of CD or BG has not been clarified, mostly according to the preferences of the operator (Wei and Ge, 2011). As the most commonly used procedure for the treatment of ONFH, CD has been used for more than 50 years, but its efficacy is still controversial (Hua et al., 2019). More hospitals are carrying out various BG techniques to treat ONFH alone and have achieved good results (Seyler et al., 2008). In recent years, attempts have been made to enhance the effect of CD with bone grafts, synthetic bone substitutes, bone morphogenetic proteins or helper cells (Martinot et al., 2020a). Among them, the cell-based CD procedure shows promising results (Hernigou and Beaujean, 2002). A large number of studies have shown that in early ONFH, implantation of autologous bone marrow aspiration concentrate (BMAC) into necrotic lesions through CD is more effective than CD alone in improving pain and hip function and reducing the number of hips that progressed to subchondral fractures (Papakostidis et al., 2016). Bone marrow contains a variety of stem cells and stromal cells with osteogenic potential, but there is a lack of standardization in the source of bone marrow, the quantification of transplantation and the processing and quantification of cells. For these reasons, it is very important to establish standardized treatment procedures for ONFH based on BG and cell-based CD.

The purpose of this study is to provide some valuable suggestions for surgeons by comparing the efficacy of CD and BG. The difference between CD combined with bone marrow transplantation (CD + BM) and CD alone was evaluated by hip pain, function and THA conversion rate. The most important thing is to quantify bone marrow and cell transplantation through subgroup analysis, so as to provide guidance for the standardized treatment of ONFH in the future.

## Materials and methods

### Search strategy

This work was conducted in accordance with the Preferred Reporting Items for Systematic Reviews and Meta-Analyses (PRISMA) Statement. PubMed, Embase, and the Cochrane Library were used to search relevant research in recent ten years (from 2012 to 2022). The following search terms were used, alone or in combination: “osteonecrosis of the femoral head” or “femoral head necrosis” or “femur head necrosis” or “avascular necrosis in femoral head” and “Core decompression” or “Bone grafting”. We did not impose any language restrictions on our search.

### Eligibility criteria

We reviewed all the retrieved abstracts and full texts. The inclusion criteria for CD and BG comparison are as follows: (1) comparative study of CD and BG in the treatment of ONFH, (2) complete information of the patients and the detailed scores and imaging data before and after treatment, (3) patient has not received other adjuvant treatment measures, such as extracorporeal shock wave, drug treatment, (4) each study was followed-up for at least two years. The inclusion criteria for CD and CD + BM comparison were similar to the above. The comparative study of CD and CD + BM was included, and the review, case report and animal experiment were excluded.

### Quality assessment

We followed the guidelines developed by the Cochrane Collaboration to assess the risk of bias in randomized controlled trials. To draw the risk assessment summary figure, we used: random sequence generation; allocation concealment; blinding of participants and personnel; blinding of outcome assessment; incomplete outcome data; and selective reporting. The bias risk assessment tool for non-randomized controlled trials also has 7 evaluation dimensions, including: confounding; selection bias; bias in measurement classification of interventions; bias due to deviationsfrom intended interventions; bias due to missing data; bias in measurement of outcomes; bias in selection of the reported result.

### Outcome measures

Two researchers independently extracted the data using standardized forms, and the third researcher then verified the accuracy of the synthesized data. The extracted data include the name of the first author, year of publication, sample size of each group, average age, ONFH grade, follow-up time and bone graft type, bone marrow dose, cell number and research outcomes. The effectiveness of the treatment of ONFH was evaluated by the improvement of hip pain and function, and the conversion rate of THA. Mean change in Visual Analog Scale (VAS) from baseline was used as the main criteria for pain. Western Ontario and McMaster University Osteoarthritis Index (WOMAC) and Harris Hip score (HHS) were used as criteria for functional changes. Most importantly, we performed a subgroup analysis of bone marrow dose and cell number. The volume of bone marrow injected into the femoral head is less than 10 mL, which is a low dose group. More than 40 mL is a high dose, and the rest is medium group. The number of cells was divided into two groups, 2–5 × 10^8^ for low dose and 2–5 × 10^9^ for high dose.

### Statistical analysis

This work was performed using Revman 5.3 software. For continuous data, the results were reflected by mean difference (MD) and 95% confidence interval (CI). Calculate the odds ratio (OR) and 95% CI for the binary data result statistics. The I^2^ statistic was used to assess heterogeneity in the assay, and value 50% or higher for high heterogeneity. When I^2^ > 50%, the random effect model was adopted, and when I^2^ < 50%, the fixed effect model was adopted. All *p* values <0.05 were considered statistically significant.

## Results

### Selection of included studies

**FIGURE 1:**
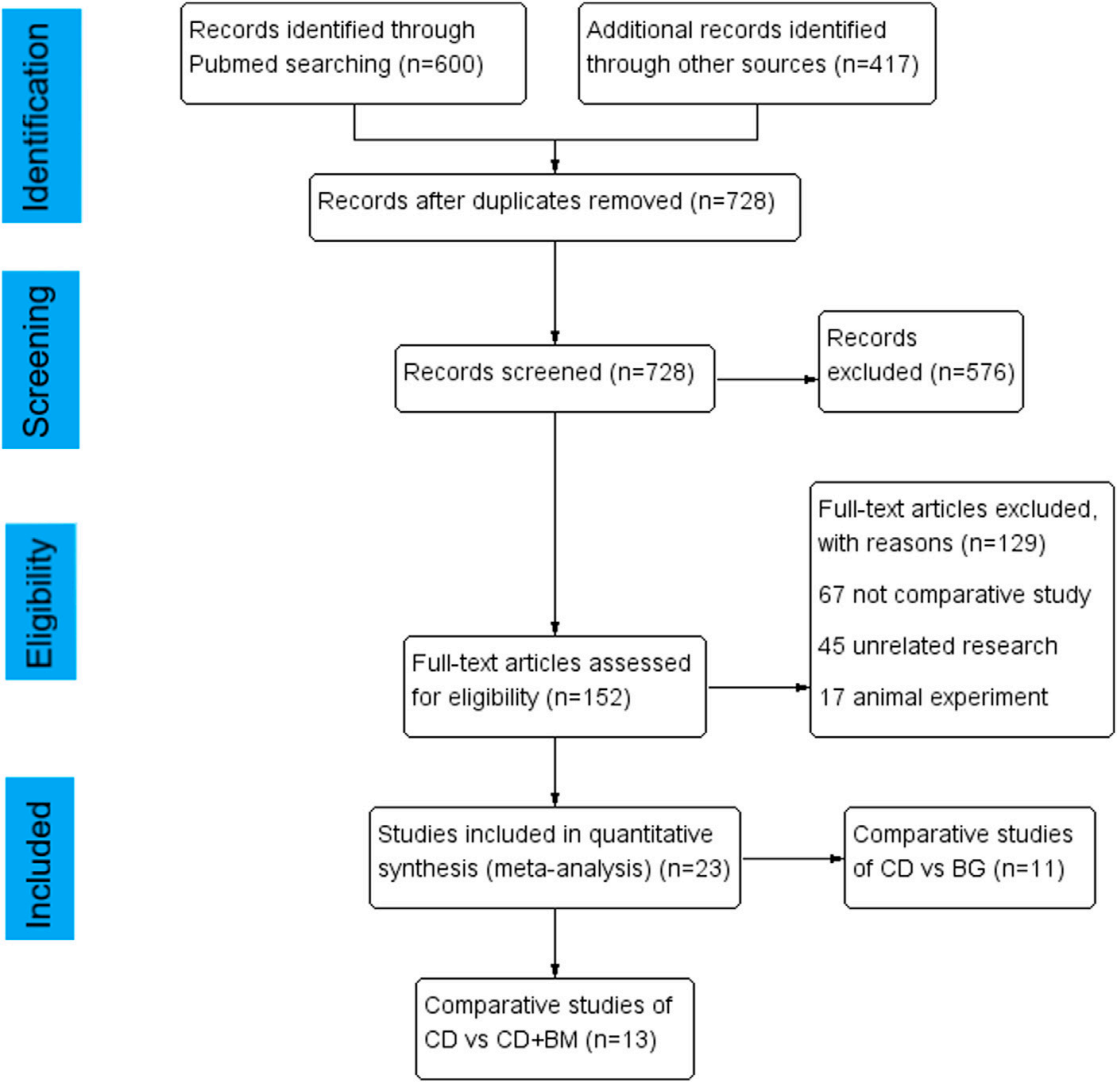
Flow diagram showing the process of inclusion and exclusion.

In total, 600 articles were obtained by searching PubMed database, 417 articles were obtained from other databases, and then all duplicate articles were deleted. After scanning the title and abstract, 576 unrelated articles were excluded. Subsequently, through the review of the full text, 23 articles were included in the study. Among them, the comparison between CD and BG includes 11 articles (Yang et al., 2010; Zhao et al., 2013; Li et al., 2016; Cao et al., 2017; Mohanty et al., 2017; Sallam et al., 2017; Hu et al., 2018; Nally et al., 2018; Lakshminarayana et al., 2019; Wang et al., 2020a; Shiravani Brojeni et al., 2020), and the comparison between CD and CD + BM includes 13 articles (Gangji et al., 2011; Sen et al., 2012; Zhao et al., 2012; Liu et al., 2013; Ma et al., 2014; Tabatabaee et al., 2015; Pepke et al., 2016; Hernigou et al., 2018a; Hauzeur et al., 2018; Kang et al., 2018; Nally et al., 2018; Martinot et al., 2020b; Li et al., 2020). The flow chart of article selection is shown in Figure 1.

### Study characteristics

Eleven studies comparing CD with BG were published between 2010 and 2020, including 821 hips of 682 ONFH patients. The sample size of included studies was greater than 20, and the average age range of patients was 30–40 years old. The majority of patients in the trials were early ONFH and were followed up for at least two years. One study used Steinberg’s diagnostic criteria, four studies used FITA staging, and the other six studies used ARCO staging. Seven studies compared CD with various BG techniques, and the other four studies compared CD combined BG group with CD control group. The evaluation of clinical results includes HHS score and THA conversion rate. There are few studies on VAS score, which are not used for analysis. Detailed characteristics are summarized in Table 1.

**TABLE 1:** Trials of CD and BG comparison.

Author (year)	Patients (male)	Hips (CD/BG)	Age (SD)	Etiology (Steroids/Alcohol)	Study type	Bone graft	Follow-up (years)	Disease stage	Outcome measure
Cao et al. (2017)	21 (16)	21/21	31 (6)	7/8	RCT	free vascularized fibular graft	3	ARCO I/II/III	HHS, THA
Hu et al. (2018)	130 (90)	65/65	40 (7)	NA	RCT	fibula fixation	4	ARCO I/II	HHS
Li et al. (2016)	39 (27)	23/24	36.5 (9)	8/23	RCT	quadratus femoris muscle pedicle	2.5	Ficat I/II	HHS, THA
Sallam et al. (2017)	61 (38)	38/33	33 (8.5)	16/0	case-control	inverted femoral head graft	7.86	Ficat I/II/III	HHS, THA
Shiravani Brojeni et al. (2020)	46 (30)	30/28	31 (6)	36/0	cross-sectional	impaction of cancellous allograft	5	Ficat II/III	VAS, HHS, THA
Wang et al. (2020a)	125 (96)	59/66	39 (10)	40/69	retrospective	iliac crest cancellous bone	4	ARCO I/II	VAS, HHS, THA
Zhao et al. (2013)	32 (18)	23/22	30 (6)	16/3	retrospective	vascularized greater trochanter	4.8	ARCO I/II	HHS, THA
Lakshminarayana et al. (2019)	46 (38)	36/40	30 (7)	12/9	prospective	CD + non-vascularized fibular graft	4.46	Ficat I/II	VAS, HHS, THA
Mohanty et al. (2017)	46 (36)	33/35	35 (7.5)	8/6	prospective	CD + fibular strut graft	3	Ficat I/II/III	HHS, THA
Nally et al. (2018)	60 (33)	47/34	39 (10)	23/0	case–control	CD + iliac crest cancellous bone	6.42	Ficat I/II	THA
Yang et al. (2010)	76 (44)	22/56	37 (5)	45/11	prospective	CD + biomaterialloaded allograft threaded cage	3	Steinberg I/II/III	HHS, THA

Similarly, Table 2 summarizes the characteristics of comparative studies of CD alone and CD + BM. A total of 13 studies from 2011 to 2020 were included, containing 920 hips of 698 patients. Except for one study that did not concentrate bone marrow, all other studies used the technique of concentrating and separating cells from bone marrow. The bone marrow of 7 studies came from the iliac crest, 4 studies from the posterior superior iliac spine, and the rest from the proximal femur.

**TABLE 2:** Trials of CD and CD + BM comparison.

Author (year)	Patients (male)	Hips (CD/CD + BM)	Age (SD)	Etiology (Steroids/Alcohol)	Study type	Bone marrow dose (source)	Mononuclear cells number	Follow-up (years)	Disease stage	Outcome measure
Gangji et al. (2011)	19 (9)	11/13	44 (2.8)	20/2	prospective	49.7 ± 2.3 mL iliac crest	1.9 ± 0.2×10^9^	5	ARCO I/II	VAS, THA
Hauzeur et al. (2018)	38 (27)	23/23	49 (3)	25/15	RCT	48.33 ± 1.16 mL iliac crest	3.5 ± 0.4×10^9^	2	ARCO III	VAS, WOMAC, THA
Hernigou et al. (2018a)	125 (78)	125/125	36 (9)	125/0	RCT	20 mL iliac crest	2.1 ± 0.5×10^9^	2	Steinberg I/II	HHS, VAS, THA
Kang et al. (2018)	100 (74)	53/53	46.5 (9)	10/38	case–control	15 mL proximal femur	2.1 ± 2.1×10^8^	4	ARCO I/II/III/IV	VAS, THA
Li et al. (2020)	31 (22)	20/21	36 (8)	19/11	RCT	1 mL posterior superior iliac spine	3×10^9^	10	Ficat II/III	VAS, WOMAC, THA
Liu et al. (2013)	34 (27)	28/27	38 (5.5)	11/17	retrospective	5 mL posterior superior iliac spine	1.6 ± 0.2×10^8^	3	ARCO II	HHS, VAS, THA
Ma et al. (2014)	39 (28)	24/25	35 (10)	26/7	RCT	1 mL posterior superior iliac spine	3 × 10^9^	2	Ficat I/II/III	VAS, WOMAC, THA
Martinot et al. (2020b)	67 (55)	24/43	42 (10)	26/12	case–control	20 mL iliac crest non-concentrated	NA	5.33	Ficat I/II/III	HHS, THA
Nally et al. (2018)	60 (33)	47/34	39 (10)	23/0	case–control	<3 mL iliac crest	NA	6.42	Ficat I/II	THA
Pepke et al. (2016)	24 (21)	14/11	44 (3.4)	NA	RCT	10 mL iliac crest	1.2 ± 0.2×10^8^	2	ARCO II	HHS, VAS, THA
Sen et al. (2012)	40 (27)	25/26	NA	14/6	RCT	2 mL posterior superior iliac spine	5 × 10^8^	2	ARCO I/II	HHS, THA
Tabatabaee et al. (2015)	28 (19)	14/14	29 (8)	19/0	RCT	58 ± 13 mL iliac crest	5 ± 2 × 10^8^	2	ARCO I/II/III	VAS, WOMAC, THA
Zhao et al. (2012)	93 (46)	44/53	33 (9)	23/18	RCT	2 mL subtrochanteric	NA	5	ARCO I/II	HHS, THA

### Assessment for risk of bias

Most RCTs generate random sequences, but do not explain the method of allocation concealment. Most of the RCTs did not specify how to implement the blinding method, which caused the risk of bias (Supplementary Figure S1). Three non-RCTs with high risk of bias in measurement classification of interventions, because these three studies intervened according to the classification of femoral head necrosis. Several experiments did not indicate whether the operation was performed by the same doctor or the same team, with implementation bias (Supplementary Figure S2).

### CD vs. BG clinical outcomes

**FIGURE 2:**
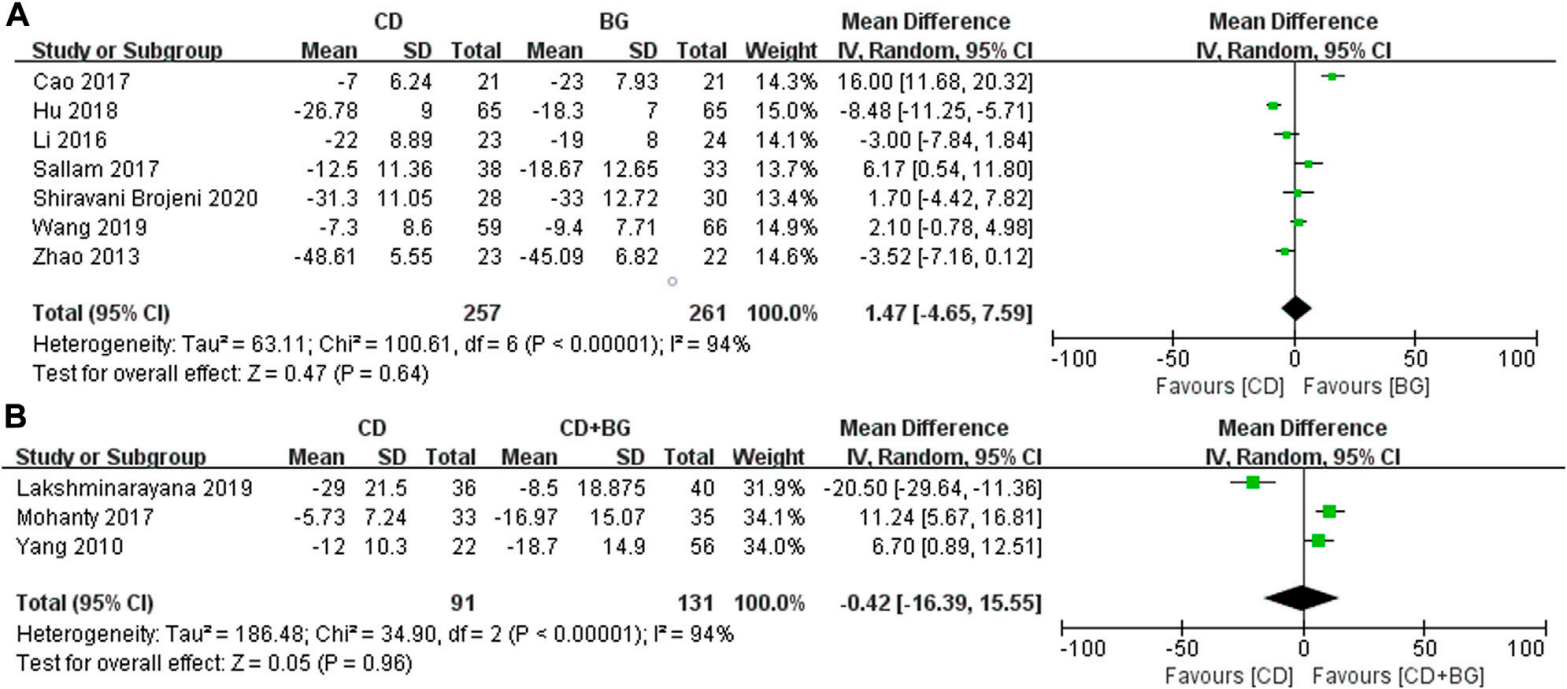
Forest plots of CD vs. BG Mean Difference (MD) on VAS scores.**(A)** MD of CD vs. BG. **(B)** MD of CD vs. CD + BG.

In order to evaluate the improvement of hip pain and function, we extracted the changes of HHS, but one study did not provide relevant data. We observed that compared with CD, BG and CD + BG did not show significant improvement, which was not statistically significant. The mean difference (MD) was 1.47 (95% CI—4.65 to 7.59; *p* = 0.64) and—0.42 (95% CI − 16.39 to 15.55; *p* = 0.96), respectively (Figure 2).

**FIGURE 3:**
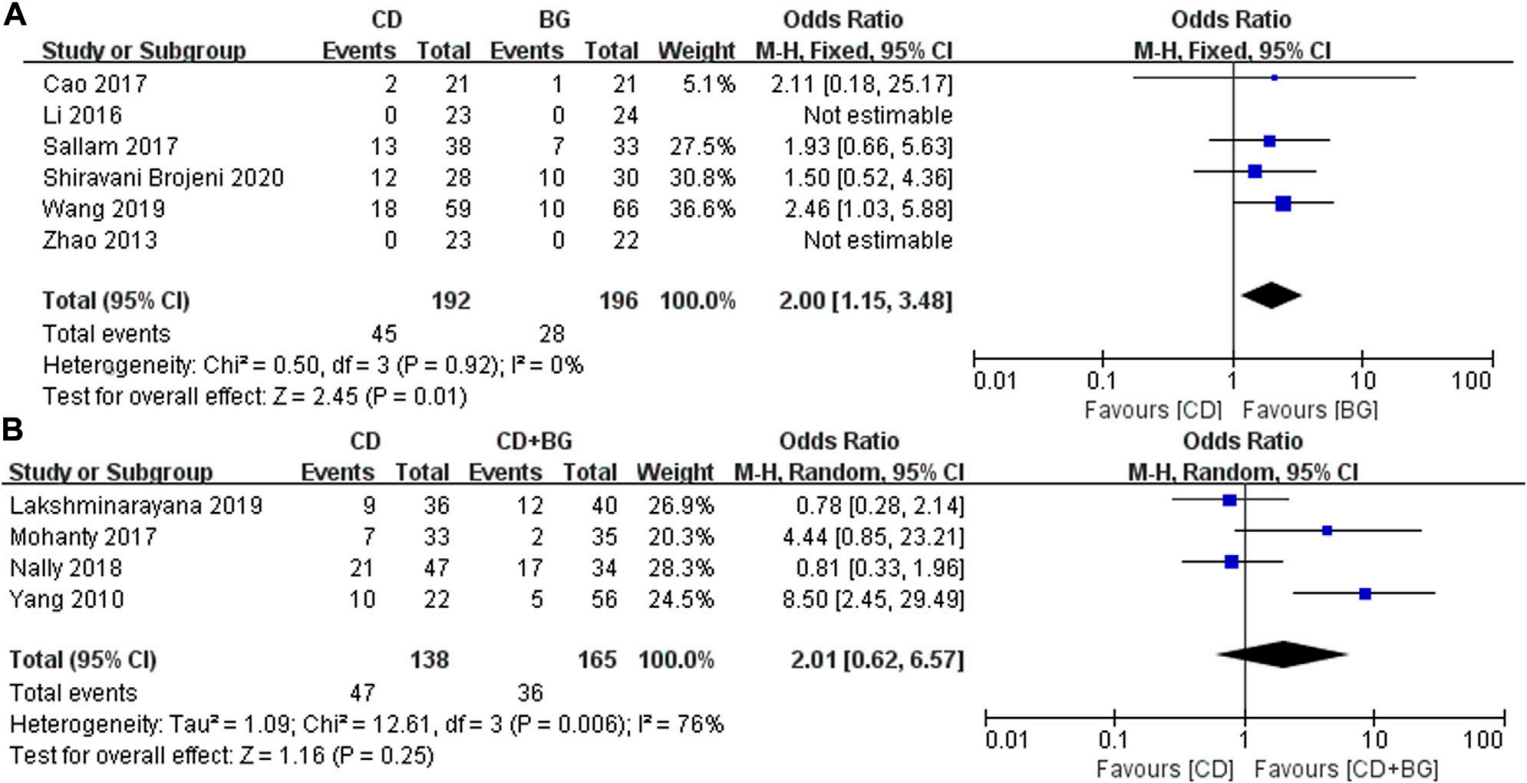
Forest plots of CD vs. BG Odds Ratio (OR) on THA conversion rate. **(A)** MD of CD vs. BG. **(B)** MD of CD vs. CD + BG.

In addition, the most concerned question is whether the surgical method can delay the progress and preserve the femoral head. The number of postoperative collapse and conversion to THA in CD group was significantly higher than that in BG Group, the difference was statistically significant, *p* values = 0.01. And no heterogeneity was found between these studies. Interestingly, the treatment of CD + BG did not show superiority, and the THA conversion rate did not decrease, *p* values = 0.25 (Figure 3).

### CD vs. CD + BM clinical outcomes

**FIGURE 4:**
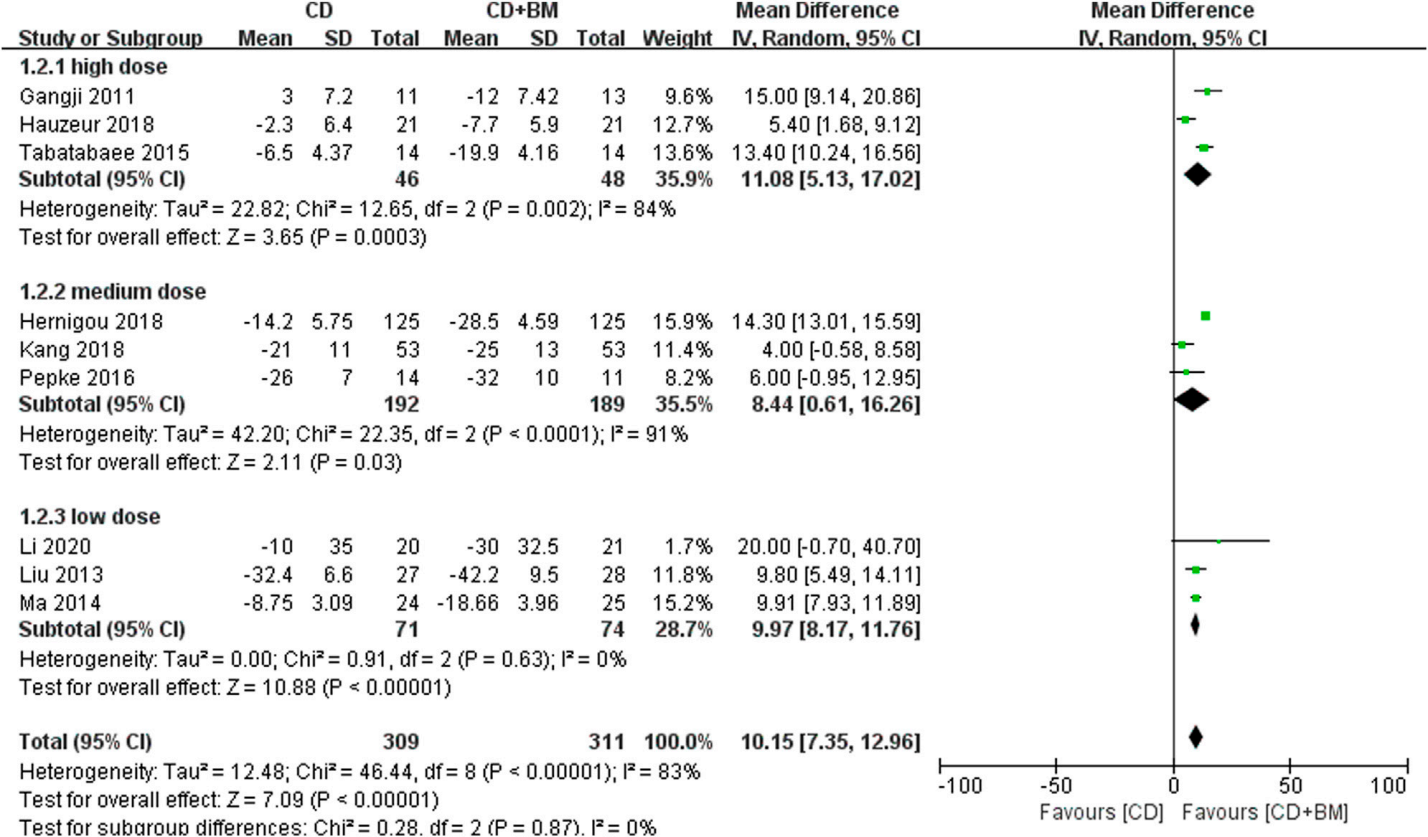
Forest plots of CD vs. CD + BM Mean Difference (MD) on VAS scores according to bone marrow dose.

First, we extracted the clinical indicators of hip pain. Nine studies used VAS to evaluate the pain efficacy of bone marrow transplantation. A total of 311 hips received bone marrow transplantation and 309 hips received CD. The MD in VAS changes in patients treated with BM significantly decreased by 10.15 (95% CI 7.35 to 12.96; *p* < 0.00001) compared with that of the controls. Heterogeneity was found in a high-dose group and a medium dose group, but did not affect the final results. Each dose could alleviate hip pain compared with CD alone (Figure 4).

**FIGURE 5:**
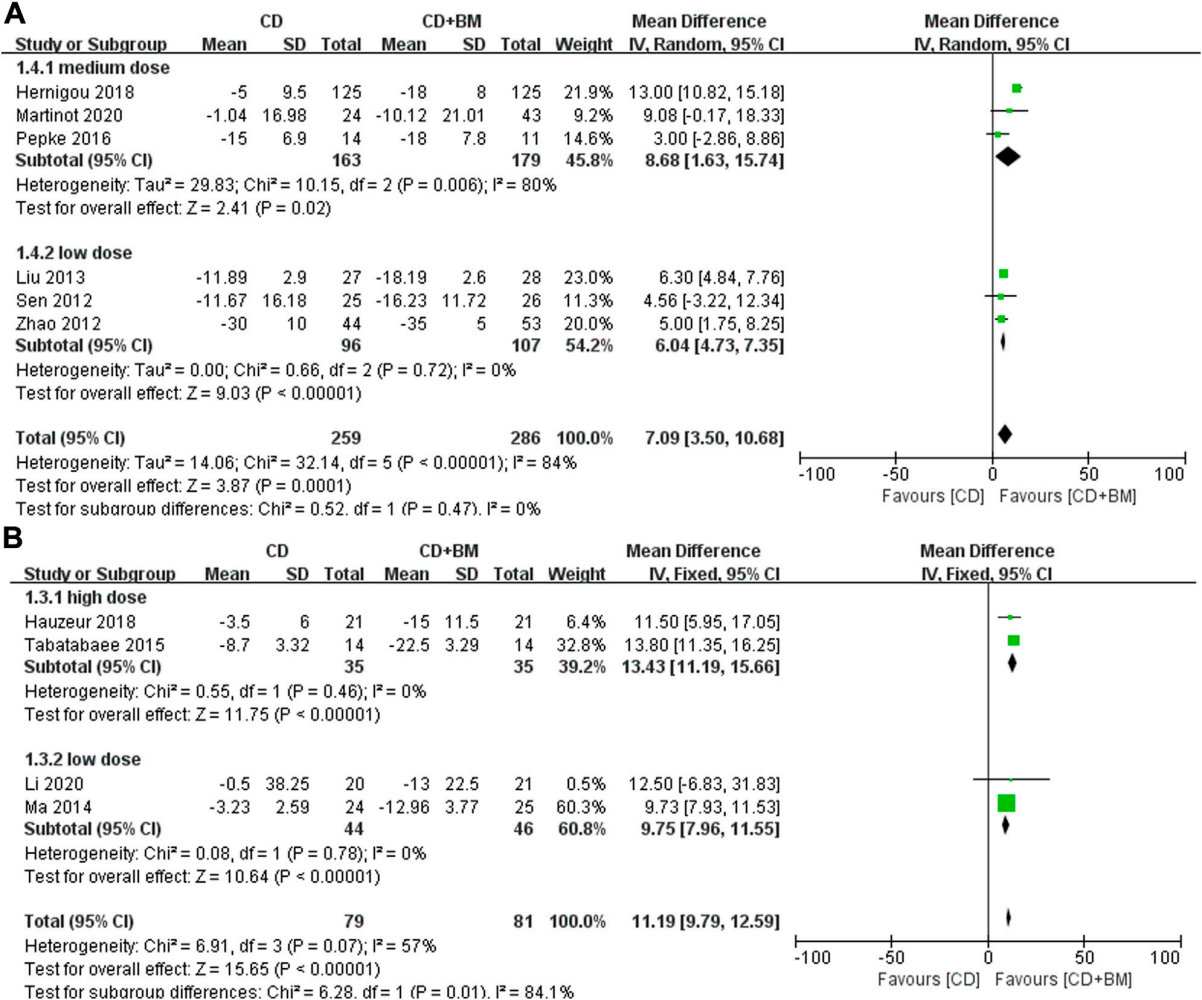
Forest plots of CD vs. CD + BM Mean Difference (MD) on HHS and WOMAC scores according to bone marrow dose. **(A)** MD of HHS scores. **(B)** MD of WOMAC scores.

Function was another clinical parameter to evaluate the hip joint in these studies. Six studies adopted HHS score and four studies adopted WOMAC score, and the data were normalized. We observed that the *p* values of both scores were very small, and the different doses were statistically significant. There was no heterogeneity between the middle dose group and the low dose group. And there was high heterogeneity between the high dose group and the low dose group (I^2^ = 84.1%), the *p*-value of WOMAC in low dose group was higher than that in high dose groups. (Figure 5).

**FIGURE 6:**
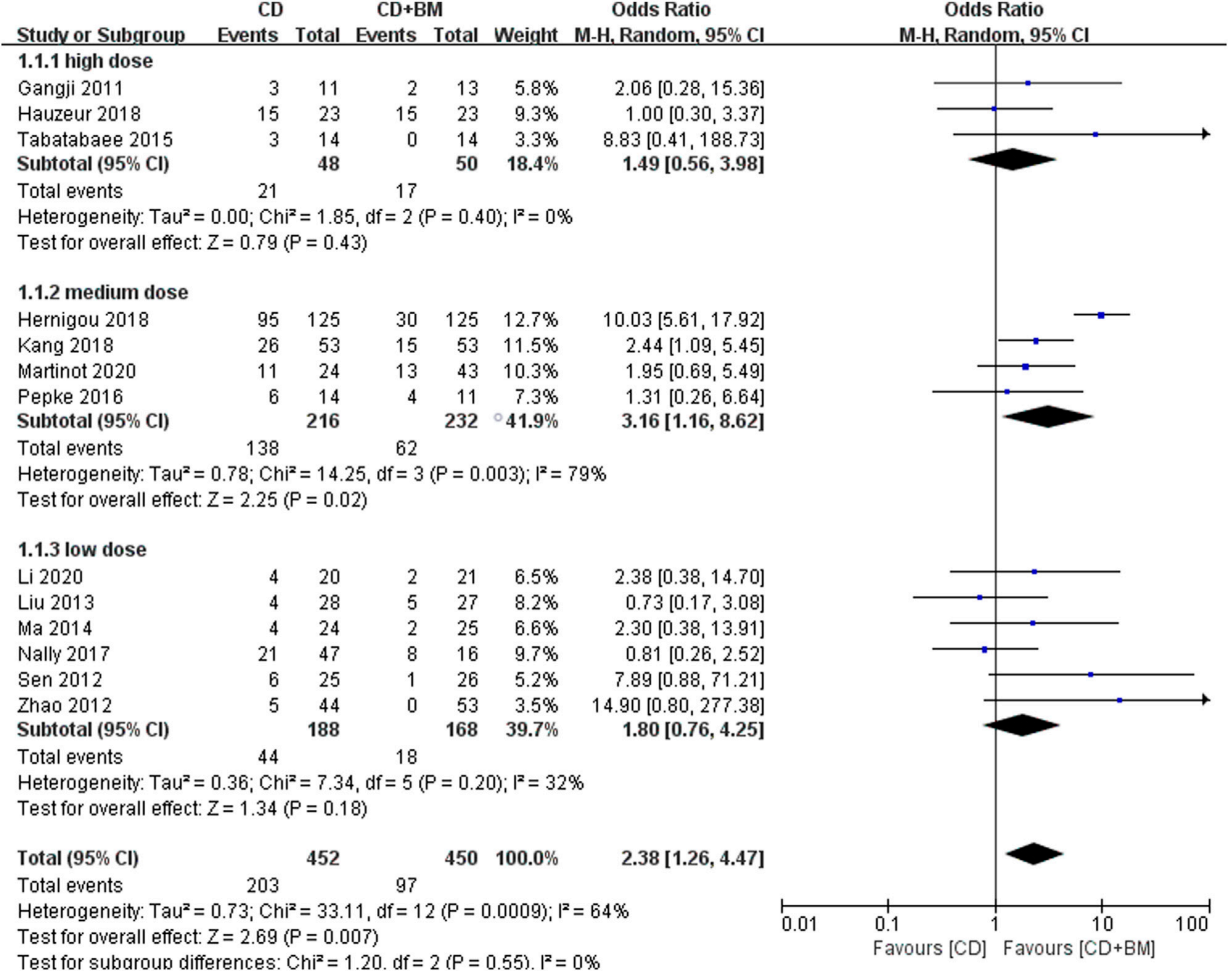
Forest plots of CD vs. CD + BM Odds Ratio (OR) on THA conversion rate according to bone marrow dose.

Moreover, all studies also counted the number of patients receiving THA after the end of follow-up. It should be noted that only the *p*-value of the medium dose group was lower and statistically significant. Excluding the heterogeneity study of Hernigou, the medium dose still showed an advantage. Overall, BM treatment can reduce THA conversion rate, OR = 2.38 (95% CI 1.26 to 4.47; *p* = 0.007) (Figure 6).

**FIGURE 7:**
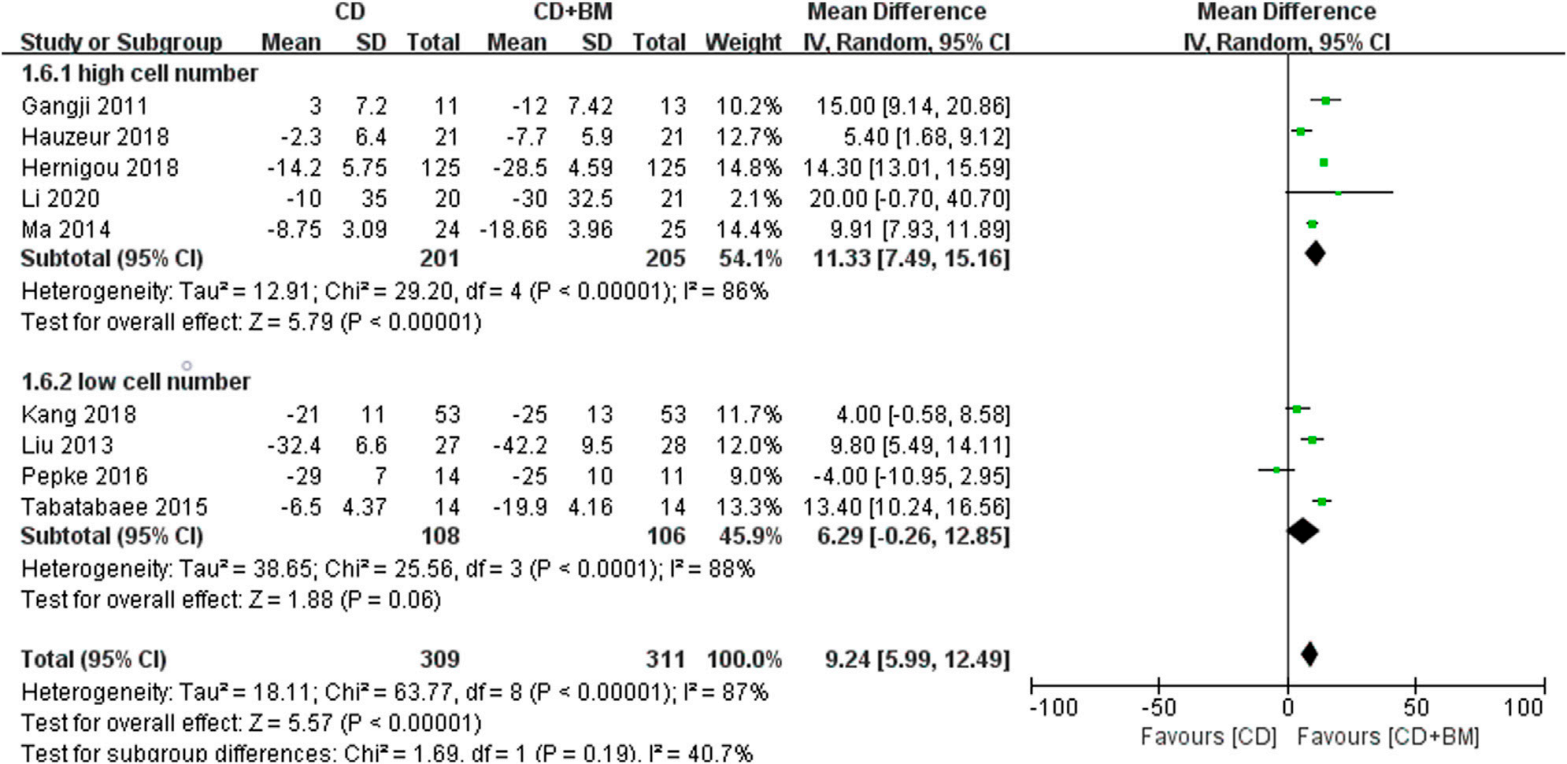
Forest plots of CD vs. CD + BM Mean Difference (MD) on VAS scores according to mononuclear cell number.

**FIGURE 8:**
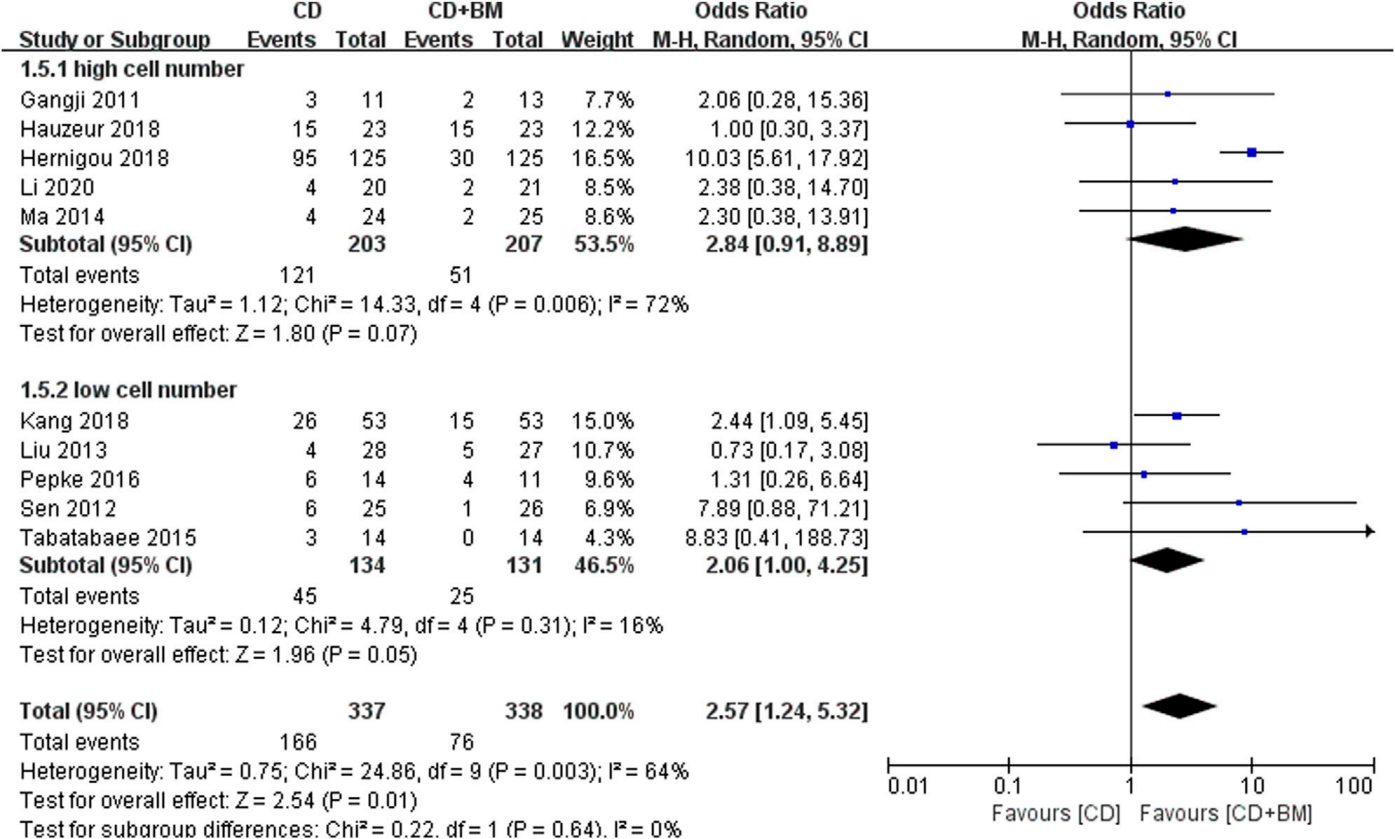
Forest plots of CD vs. CD + BM Odds Ratio (OR) on THA conversion rate according to mononuclear cell number.

Finally, VAS score and THA conversion rate were extracted to subgroup analysis of different cell numbers. The change of VAS MD in the high cell number group was statistically significant 11.33 (95% CI 7.49 to 15.16; *p* < 0.00001). The *p*-value of the low cell number group was close to 0.05, and the changing MD was 6.29 (95% CI -0.26 to 12.85; *p* = 0.06) (Figure 7). In THA conversion, there was no heterogeneity between the two groups of different cell numbers (I^2^ = 0%), and the *p*-value of the low cell number was closer to 0.05 (Figure 8).

## Discussion

In the early stage of trying to preserve the native joint, the most effective treatment for osteonecrosis continues to be debated. Although CD can reduce intraosseous pressure and promote the formation of blood vessels and new bone, CD alone seems to be no longer recommended (Rajagopal et al., 2012). Martinot found that augmented CD can improve the survival rate and produce better clinical results through two-year follow-up (Martinot et al., 2020a). Our meta-analysis first compared the efficacy differences between CD and BG. We found no significant difference in HHS score, but the number of patients receiving THA after CD was significantly higher than that after BG. This may be related to the fact that BG can provide structural support and play a supporting role. There are a variety of BG options, including non-vascularized or vascularized autologous bone from iliac crest, fibula or femur, as well as allogeneic and synthetic bone (Kim et al., 2005; Xie et al., 2019). Of the studies we included, four used fibula, two used iliac crest, and allogeneic bone. Some small-scale experiments have compared the advantages and disadvantages of different bone transplantation techniques, but there is no high-quality randomized controlled trial to clearly recommend which one (Tu et al., 2017). Wan compared four bone grafts, including: free fibular graft, free vascularized fibular graft, autologous iliac bone andβ-tricalcium bioceramics phosphate graft. He believes that bioceramics graft have the advantages of short operation time and less blood loss (Wan et al., 2022). BG is sometimes also performed as an ancillary procedure for CD and as a scaffold for new bone formation. But to our surprise, Lakshminarayana’s study did not favor combined fibular graft, and both imaging and clinical showed faster progress (Lakshminarayana et al., 2019). The results of our meta-analysis also did not support CD combined with BG as a potential treatment. We believe that although BG provides bone support, the clearance of necrotic bone and the amount of filled bone will affect the postoperative effect. In addition, BG has the disadvantages of long operation time and large amount of bleeding, which is described in Wang’s experiment (Wang et al., 2020a). In particular, vascularized BG is more difficult to achieve technically and has more postoperative complications (Gonzalez Della Valle et al., 2005). Therefore, in view of the diversity of BG treatment at present, we do not recommend BG as the routine treatment of ONFH.

The most important finding of this meta-analysis is that core decompression combined with bone marrow cell transplantation can reduce pain, improve function and the long-term survival rate of hip joint. 203 of the 452 hips treated with CD progressed and underwent THA, but only 97 of the 450 hips in the CD + BM group underwent further surgery. Our study includes randomized controlled trials and non-randomized controlled trials, and selection bias cannot be avoided. However, compared with other analyses, we included more studies and only considered the factors of bone marrow cells, excluding the interference of other adjuvant treatment measures (Mao et al., 2020). Recently, cell-based CD enhancement has attracted extensive attention, but some limitations hinder their use. These limitations include the lack of evidence on the ideal cell source, the lack of methods to optimize the harvesting and processing of cells, the number and methods of transplantation and delivery of cells, etc (Talathi and Kamath, 2018). In recent ten years, most of the transplanted cells come from autologous bone marrow puncture concentrate, and platelet rich plasma from peripheral blood remains to be studied (Aggarwal et al., 2021). After the autologous bone marrow was directly aspirated from iliac crest or posterior superior iliac spine, the concentrated solution was obtained by centrifugation. However, the dose of autologous bone marrow puncture concentrate varies greatly in different experiments, and no consensus has been reached. We performed a subgroup analysis of the dose of bone marrow aspiration concentrate and concluded that 20 mL concentrate could best improve pain and function and reduce the rate of femoral head collapse. The volume of stage 3 femoral head necrotic lesions measured from CT, MR images and gross specimens was about 22 cm^3^, which was consistent with the optimal injection volume (Hu et al., 2015). Because the current technology is still limited to direct injection into the necrotic area, there is the possibility of leakage at high doses. Although the high dose did not show more complications, we believe that it still has the risk of increased intraosseous pressure and blood stasis. In Nally’s study, low-dose cell assistance did not show advantages (Nally et al., 2018), and low-dose was still mainly used in combination with other BG techniques to enhance the efficacy (Ma et al., 2014; Li et al., 2020).

Rastogi found that the effect of mononuclear cell injection isolated from bone marrow was better than that of untreated bone marrow injection through at least two years of follow-up (Rastogi et al., 2013). Therefore, mononuclear cell obtained by centrifugation of bone marrow have better therapeutic effect. Bone marrow mononuclear cells contain hematopoietic and endothelial precursor cells, stem cells and osteoblast progenitor cells, which play a role in capillary blood supply and osteoblast formation at the site of bone necrosis (Mao et al., 2013). Previous reviews have directly described bone marrow mononuclear cells as stem cells, which is inaccurate (Wang et al., 2020b). In our meta-analysis, ten studies counted the total number of mononuclear cells contained in bone marrow, and four studies made further statistics on fibroblast colony forming units. Because of the limited number of studies, it is difficult to conclude that stem cells play a major role. We only performed subgroup analysis on the number of mononuclear cells, and the results showed that cells of 10^9^ magnitude were more beneficial. Our conclusion is similar to that of Mao et al., who believe that high magnitude cells can obtain better long-term benefits (Mao et al., 2020). Hernigou also recommends 5 × 10^7^ mononuclear cells as the minimum critical number. Current studies are larger than this number, in which the number of stem cells is 10^6^–10^7^. In addition, due to the significant reduction of monocyte precursor cell concentration in bone marrow of patients with alcoholism and steroid use, it is worth considering whether allogeneic cell therapy will be a better option (Hernigou et al., 2018b). Because adipose tissue is easy to obtain and is a good source of stem cells, adipose derived allogeneic cells may be a direction of future research (Wyles et al., 2015).

## Conclusion

In conclusion, although there are many BG surgical methods, there is no consensus on which one to choose. The enhancement of cell-based CD program has a positive impact on hip pain, function and reduction of THA transformation. 20 mL bone marrow aspiration concentrate and 10^9^ magnitude bone marrow mononuclear cell injection may be a good choice. This provides a clearer guidance for the treatment of femoral head necrosis in the future.
