# Supplementary figures and images for: Comparison of current treatment strategy for osteonecrosis of the femoral head from the perspective of cell therapy
Source: Front Cell Dev Biol. 2023 Mar 22;11:995816. doi: 10.3389/fcell.2023.995816 (PMC10073660; doi:10.3389/fcell.2023.995816)

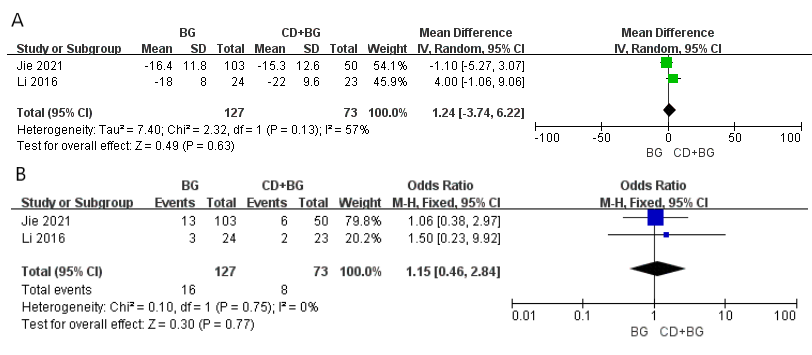

Supplement: Supplementary file 1 [file Image3.TIFF]

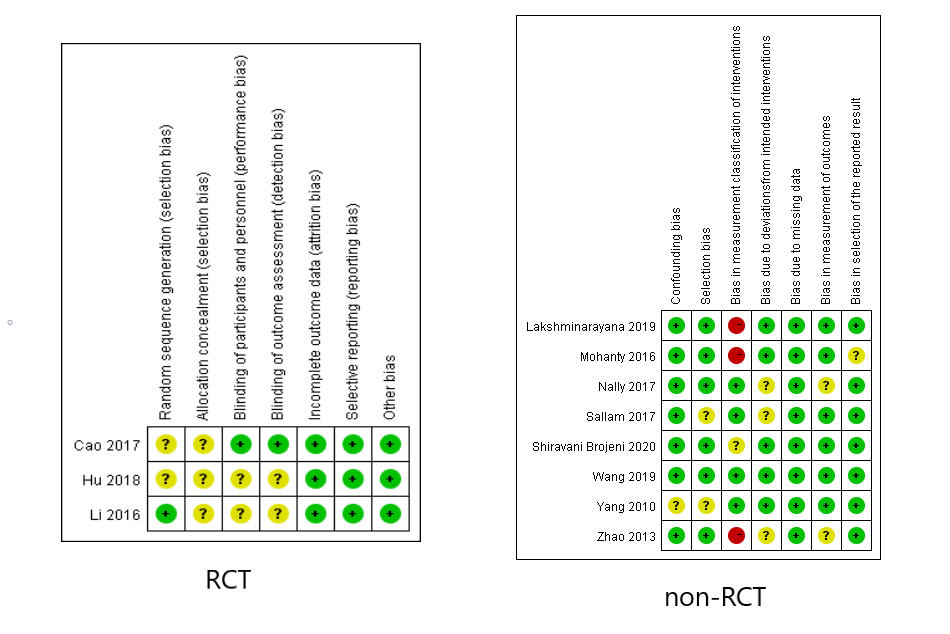

Supplement: Supplementary file 2 [file Image1.TIFF]

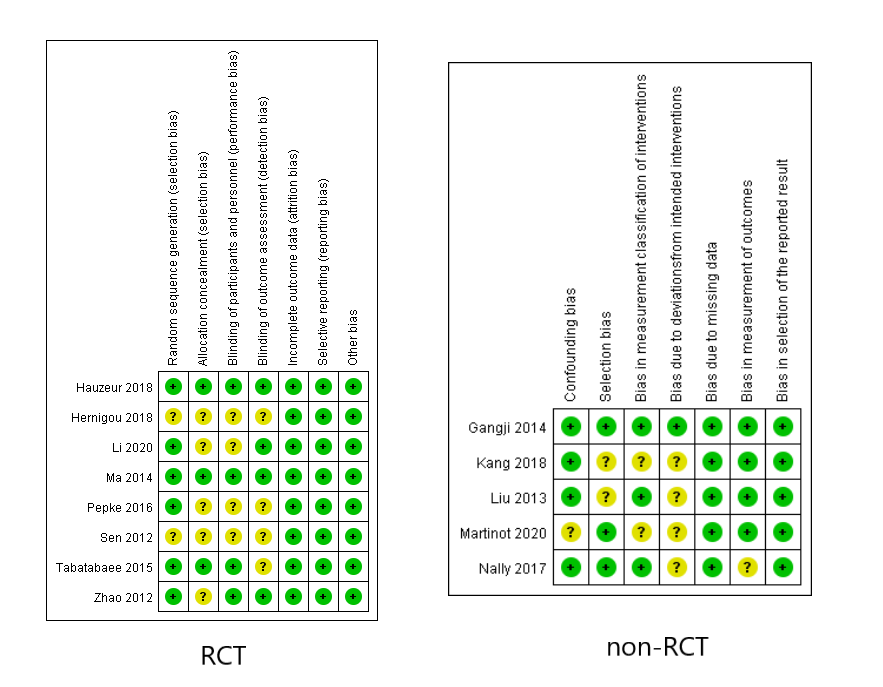

Supplement: Supplementary file 3 [file Image2.TIFF]
